# Canine Vaccination—A Survey of Owner Attitudes and Adherence to Vaccination Protocols

**DOI:** 10.3390/pathogens15070678

**Published:** 2026-06-26

**Authors:** Katrina Warnes, Daniel S. Mills, Andrew S. Cooke, Stefan H. Millson, Simon R. Clegg

**Affiliations:** 1 Joseph Banks Laboratories, Department of Life Sciences, School of Natural Sciences, University of Lincoln, Green Lane, Lincoln LN6 7DL, UK; 2 Wanderdog-Dog Training & Behaviour, Giek, 1319 BN Almere, The Netherlands

**Keywords:** dog, vaccination, anti-vaccination, over-vaccination, canine immunization

## Abstract

Vaccination is one of the most important measures for infectious disease control. Recently, media-generated concern about vaccine-associated adverse effects has produced a rise in both human and animal “anti-vaccination” movements. This study aimed to understand factors involved in dog owner vaccination decisions and explore whether there has been an increase in titer testing. An online survey targeting dog owners received a total of 2585 responses, which showed 79% of respondents had their dogs vaccinated in the last 12 months. A few owners had never vaccinated their dogs, and 13% of owners used titer testing prior to booster vaccinations. The factors with the strongest positive predictors for vaccination were requirements by third party services (e.g., kennels) and, for a negative response, lack of time. For respondents that had not vaccinated, the factors with the strongest predictive powers to determine if they titer test were education/working in the veterinary industry for a positive response and not having heard of negative side effects after vaccinating for a negative response. Overall, no evidence was found that a rise in anti-vaccination attitudes was pervasive in dog owners; however, the study shows that the veterinary profession has work to do to ensure herd immunity is maintained within dog populations.

## 1. Introduction

Vaccination has been hailed as one of the “greatest contributions to global health of any human intervention” given its success in controlling and eradicating diseases [1]. Many diseases have been eradicated through the use of vaccination, with smallpox being the most famous [2], but also huge reductions in potentially fatal companion animal diseases, such as canine distemper and parvovirus, have also been seen through successful vaccination programs [3]. Successful rabies vaccination programs of wildlife have been implemented in various parts of the world, such as in northern Europe, where use of an oral bait vaccine has led to the eradication of rabies in this region [4,5].

However, in 2019, the World Health Organization (WHO) named “vaccine hesitancy” as one of its top ten threats to global health [6]. Vaccine hesitancy is defined as “the reluctance or refusal to vaccinate despite the availability of vaccines” [7]. With the apparent control of many serious diseases, thanks largely to mass vaccination, has come public complacency and the rise of an “anti-vaccine movement” [8]. This was evident during the recent COVID-19 outbreak, although the reasons behind this are not well understood [9,10,11]. Subsequently, there has been an increase in the number of people declining vaccination for themselves and their children, potentially due to fear of vaccine-associated adverse effects (VAAEs) and the belief that vaccines may cause more harm than good [8]. The infamously incorrect link between the measles, mumps, and rubella (MMR) vaccine and autism [12] has furthered this effect, and this resulted in tens of thousands of parents not giving their children the MMR vaccine, which in turn has led to outbreaks of diseases that were once considered virtually “eliminated” [13].

The veterinary sector has recently seen a “record decline” in the number of pets being vaccinated in the UK, with the number of dogs receiving primary vaccinations falling 18% over the past three years and only a third receiving regular booster vaccinations [14]. This reduction might reflect the same hesitancy seen for human vaccination and increased fear regarding the safety and efficacy of pet vaccinations. In recent years, vaccination protocols for dogs have been the subject of great debate [15] and frequent changes. Canine core vaccinations are defined by the World Small Animal Veterinary Association (WSAVA) as those vaccines that “All dogs and cats, regardless of circumstances or geographical location, should receive” and include canine parvovirus (CPV), canine distemper virus (CDV), and canine adenovirus (CAV 1 and 2) [16,17]. In the UK, leptospirosis has also been added to these core vaccines due to its zoonotic potential [18], and, in the USA, rabies has been added and is required by law in many states [19].

Until recently, canine vaccines were licensed with a minimum duration of immunity (DOI) of only one year, and so vets typically vaccinated dogs with every vaccine annually [16]. However, there were increasingly frequent reports of mild to very serious VAAEs, and so, in response to public and media pressure, the WSAVA formed the Vaccination Guidelines Group (VGG) to produce vaccination guidelines based on evidence-based research with global applicability [16,17]. The WSAVA recommended that for adult dogs, core vaccines should be administered no more often than triennially (although this depends on the vaccine used), and most canine vaccines now reflect this three-year recommendation [20]. However, this is a minimum DOI, whereas for most core vaccines, serological and challenge studies have demonstrated protection for considerably longer [21]. Evidence also suggests that environmental boosting through natural exposure can help keep immunity levels high for dogs [19].

The WSAVA guidelines also recommend the use of triennial serological titer testing for core vaccines CPV, CDV, and CAV 1 and 2 to assess seropositivity and the requirement for core vaccine boosters [16,17,20]. However, titer tests cannot be used for leptospirosis or rabies; therefore, dogs whose geography or lifestyle places them at risk of exposure to leptospirosis or who regularly travel must still receive annual re-vaccination to leptospirosis to stay protected and triennial vaccination to rabies to stay within legal requirements [20].

In 2019, the People’s Dispensary for Sick Animals (PDSA) raised concern in its Animal Wellbeing (PAW) Report that since 2016 there has been a steep decline in primary puppy vaccination uptake, leaving them at significant risk of contracting viral and bacterial diseases “owing to the incomplete ability of their immature immune system” [14,22]. In its survey, the PDSA found 72% of dogs received primary vaccines, which is a marked reduction from 88% in 2016, and it also showed a reduction in dogs that were regularly receiving booster vaccinations (78%, down from 82%) [14]. Although there is no data that states what percentage of dogs requires each vaccine to create herd immunity for control or eradication of each core disease, it is reported that coverage of 75% is required [16]. The PDSA reported that the main reasons for the decline in pet vaccination (including cats and rabbits) were that it is too expensive (17%), not necessary (16%), or that their pet finds going to the vet stressful (13%) [14].

There are limited studies investigating potential reasons why there has been a drop in vaccine uptake. Recently, German researchers reported that a wide number of different factors were associated with owner compliance to vaccination of dogs within German national vaccination guidelines, including kennel visits and veterinarian advice [23]. Prior to this, there were two studies investigating vaccination rates in cats. These aimed at understanding factors involved in cat owners’ decisions to vaccinate and their knowledge regarding feline vaccinations [24,25]. Thus, the aim of this study was to develop a greater understanding of the factors involved in dog owners’ opinions towards vaccination of their dogs internationally and to explore whether the decline in vaccination is complemented by an increase in alternative methods, such as titer testing.

## 2. Materials and Methods

### 2.1. Ethical Note

This study was approved by the University of Lincoln ethics committee (reference number 2217). Data was obtained in compliance with general data protection regulation (GDPR) requirements, and owner consent was collected at the start of each survey with an opt-in system. All data collected was anonymous, and no personal contact data was requested.

### 2.2. Dog Owner Recruitment

A questionnaire was generated using Qualtrics software (Qualtrics Provo, UT, USA, 2020) to recruit dog owners within the UK and internationally via the social media platforms Facebook and Twitter. Multiple dog-focused groups were selected to share the questionnaire and generate a snowballing effect where participants further shared the survey on various personal pages or dog groups for a three-month period between 10 March and 10 June 2020.

### 2.3. Survey Design

The questionnaire consisted of 35 questions split into four sections (see Supplementary file S1). The first section requested demographic information regarding the owner. The second section requested information about a single dog that they own, avoiding complications regarding answering for multiple dogs. The third section requested information regarding the dog’s history of vaccination and the owner’s attitudes towards vaccinations, including alternative methods. The last section requested information on their dog’s other health care measures, such as diet, in part to deter the respondent from a focus on and potential bias towards vaccinations. All items were closed, requiring selection from multiple choice or binary yes/no answers, although some also included an optional “other” free-text box for elaboration. In addition, UK respondents were presented with a map of the UK to allow them to indicate which region they lived in.

### 2.4. Statistical Analysis

After collation of simple summary descriptions, the binary logistic regression analysis function in Minitab 19^®^ Statistical Software (State College, PA, USA: Minitab, Inc. 2019) was used to model the data. A stepwise backwards model selection process using all available potential factors was performed using the Akaike information criterion using a significance level of 0.05.

The aim was to answer the following research questions:

• RQ1. What factors significantly predict whether someone is less likely to have vaccinated in the past 12 months?
• RQ2. What factors significantly predict whether someone who has not vaccinated in the past 12 months uses titer testing?

Analysis for the second question was performed on a reduced data set only including respondents who had selected “no” for the question “vaccinated in the past 12 months.” Model assumptions were checked, most notably multicollinearity and outliers. Outliers were determined through analysis of the residuals/fit. A goodness-of-fit test was performed to test model fit, indicating a good fit for both models Q1 (Hosmer–Lemeshow: chi-square = 2.74, df = 8, *p*-value = 0.950); Q2 (Hosmer–Lemeshow: chi-square = 10.54, df = 8, *p*-value = 0.229).

The statistical metrics *p*-values, chi-square, odds ratio (OR), and confidence intervals were used for interpretation. Reference categories were selected by using the response options that had the highest proportion of respondents. An OR > 1 showed an increased expected probability and an OR < 1 showed a decreased expected probability in relation to their respective reference categories.

## 3. Results

### 3.1. Survey Response

A total of 2993 questionnaires were completed, while 385 were removed as incomplete surveys, 14 were removed as participants were under 18 years of age, 7 were removed for duplicate responses, and 2 were removed for non-consent to take part. This left 2585 responses for initial analysis.

There was a distribution across age groups, with the 18–39 and the 40–59 age group each making up around 40% of the participants, respectively (Table S1), although the female respondents were highly overrepresented, making up 95% of participants. Different levels of education were all well-represented within the participant cohort, with 39% having a degree, 18% having a higher degree, 10% with A or AS levels, and 11% with vocational awards. The remaining participants had various other educational qualifications (Table S1). Employment status varied, with 46% working full time, 13% employed part time, and 16% self-employed. The remainder were students or unemployed for various reasons (25%). Salaries were equally variable, with 26% having an income under GBP 20,000 and 23% earning between GBP 21,000 and GBP 40,000. The remaining 28% earned over GBP 41,000, and 23% did not want to reveal their salary data (Table S1).

Although the study is heavily UK-based, with 49.71% of participants, there were 114 participants from the USA and Canada, as well as others from Europe (19) and Australia and New Zealand (*n* = 21) (Figure S1). Within the UK, there was a fair spread across most parts of the UK but only a small number of respondents (*n* = 5) from Northern Ireland. In terms of education, 56.8% (1470) respondents had a university education, with 1003 (68.2%) having an undergraduate degree and 467 having a higher degree.

Dog age was also varied, although most participants owned dogs that were under three years old (38.95%); this is the age when animals should be receiving early vaccinations and worming/flea treatments as their immune system is still developing. A mix of pedigree and cross-bred animals was included (Table S1). Owners had obtained their animals from a wide variety of different places, with breeders (30.6%) and dog shelter adoption (33.6%) being the two most popular. Most animals had received some level of training, but most of this was self-taught (81%) although often in conjunction with other methods. Furthermore, 1.1% of animals were not registered with a veterinarian, 51% were uninsured, 21.9% were not neutered, and then 6.8% were not microchipped (Table S1).

Of the dogs included in the study, 21% of the animals were not vaccinated (Table 1, Figure S2), 61.8% of animals were receiving yearly vaccination, and 2% were never vaccinated. Although the majority of people in the study did vaccinate their pets, there were still a wide variety of reasons given for non-vaccination: 17.9% of people claimed they did not vaccinate their pets over concern of adverse health effects in the animal and 9.9% felt that vaccination was not required. Other less common reasons included cost (2.66%), stress to the animal when visiting the vet (2.2%), and a lack of contact with other dogs (2%) (Figure S2). Time to take the pet to the vet for the vaccination appeared not to be a big issue with participants (0.8%) (Figure S2).

The reasons why people vaccinate their pets regularly are summarized in Table 2. The most common reason was related to concern over the severity of the disease the dog could catch and the importance of protecting against it, while 48.2% highlighted the importance of veterinary advice in shaping their decision and 15.7% had vaccination included within their plan with their vet. Pressure from third parties, such as dog kennels or catteries, to show proof of vaccination to allow their animals to stay was also relevant.

Interestingly, 69% of the respondents to the questionnaire had heard about negative reactions to vaccination, with 16.4% of people claiming to have encountered them themselves (Table 2).

To further understand details regarding negative reactions people had encountered or heard of with vaccines, the respondents were asked to name conditions and signs they had heard could be linked to vaccination. The more common answers were allergies (37.5%), lethargy (33.4%), soreness at the injection site (25.5%), seizures (28.4%), autoimmune problems (24.3%), lack of appetite (22.8%), diarrhea (21.2%), death (19.7%), nausea, (19.1%), fever (19.1%), and face or limb swelling (11%), as well as behavioral issues, including aggression and reactivity (6.7%), (Table 3). Autism was referred to by 3% of respondents. All negative effects reported by participants are reported in Table 3.

Veterinarians were a common source of information for 83.7% of people, with personal research and the internet also popular (42.5% and 18.8%, respectively) (Figure S3). Other animal professionals, such as behaviorists, breeders, trainers, or rescues, were an important source of information for 12% of respondents. Interestingly, 86 dog owners reported that they had never looked to seek any advice.

Other methods, such as detox, have been suggested instead of vaccination, and 20.4% of the respondents had heard of it, with 28.6% of these people having tried it.

For comparative purposes, it is worth noting that flea treatments were used by 69.8% and worming treatments by 74.6% of participants, with 6.7% using unknown alternative methods for flea and worm treatments. In addition, 67.9% of participants had heard about negative effects related to one or both of these treatments, and 16.7% claimed to have actually experienced these. These were not examined further in the survey, though.

Along with vaccination, anti-flea and anthelminthic treatments are commonly used to protect animals from infestations and infections, respectively. Therefore, it was assessed if those who do not vaccinate do not take other preventative control methods. Reasons for anthelminthic and flea treatments were very similar to those seen for vaccination uptake, with 51.1% occurring during a veterinary consultation and 57.6% doing it out of concern for their pet’s health (Table S2). Interestingly, 55.4% engaged in flea and worm treatment to prevent infestation within the house, which may be due to the biting behavior and general annoyance caused by fleas or through knowledge of zoonotic risk of parasites (Table S2). But again, surprisingly, 12.8% of participants did not treat their pets regularly (Table S2), although how this corresponds to the animal’s lifestyle is unknown.

Interestingly, there was a wide variety of different diets being used by the participants of this study to feed their pets, including dry and wet food. Raw food was used by a large percentage of people for their pets, too (homemade by 15.2% and commercial by 22.8%) despite concerns regarding balanced diets and the risk of zoonotic infections (Table S2). Advice was again from veterinarians (52.8%), veterinary nutritionists (21.5%), or their own research (53.9%). Furthermore, 38.5% obtained nutritional information from the internet, which is unsurprising, and 28.5% obtained information from their friends or fellow dog owners (Table S2). Again, professionals like behaviorists, groomers, and breeders provided nutritional information for 15.7% of participants, and, again, it may be pertinent to offer appropriate training to these professionals as part of their continued professional development (Table S2).

The most interesting factors associated with the response “vaccinated in the past 12 months” were answering yes to “Lack of time to go to the vets” (*n* = 20/2585; OR 8.5; 95% confidence interval (CI) 1.9–37.7) and answering no to “flea and worm regularly due to products being part of pet plan” (*n* = 297/2581; OR 7.3; 95% CI 2.3–22.5) due to their significant association with a negative response to the target question (Table 4). Additionally, answering yes to “Vaccinate regularly due to requirement of third party-proof” (*n* = 657/2585; OR 0.3; 95% CI 0.2–0.5) was associated with a positive response to the target question.

### 3.2. Factors Predicting Whether an Owner Who Did Not Vaccinate in the Previous 12 Months Uses Titer Testing

Based on AIC model selection, multiple factors were associated with “titer test and vaccinate when required” (Table 5 and Table 6), the most interesting of which are answering yes to “Education or work in vet or nutrition industry” (*n* = 9/540; OR 8.5; 95% CI 1.3–53.9), answering yes to “feeding cold pressed/air dried/freeze-dried dog food” (*n* = 66/540; OR 4.0; 95% CI 1.9–8.5), and answering yes to “advice received regarding nutrition from a nutritionist” (*n* = 177/540; OR 3.6; 95% CI 2.2–6.2) due to their significant association with a positive response to the target question. Note that the factor “Education or work in vet or nutrition industry” was chosen to be included despite the small sample size, and this result may vary with a larger sample; of the responses, six answered that they titer test, while three answered that they did not titer test. Additionally, answering no to “heard of negative effects after vaccinating” (*n* = 479/543; OR 0.1; 95% CI 0.03–0.5) was associated with a negative response to the target question.

## 4. Discussion

This study was designed to assess vaccination in pets and why or why not owners choose to treat their pets with the relevant vaccines and other treatments. The results showed that 79% of dogs in the study were vaccinated in the past 12 months. In comparison, a recent German study regarding dog vaccination compliance found that only 47% of dogs were vaccinated annually to recommended levels (including leptospirosis [23]); however, the research focused only on German dog owners, whereas respondents of this current study were global but heavily biased to Europe and the USA.

Vaccination protocols may differ significantly between countries. Though WSAVA guidelines are meant for international guidance, they acknowledge that some countries may have differing non-core vaccination requirements depending on disease risk level and legal requirements. This study, for example, found that there was a large difference between numbers of those who vaccinated in the previous year between the USA/Canada (89%) and the UK (70%). In the UK, there are no legal obligations to vaccinate any dog (requirements exist only for international travel and for entry into kennels and some dog shows), whereas in the USA (where rabies is endemic) annual vaccination is legally required in many states, even for products licensed with a 3-year duration of immunity (DOI) [16]. The WSAVA guidelines, however, are not mandatory [16,17], and there are still vet practices in the UK and abroad that do not follow these guidelines and still encourage core annual re-vaccination regardless of the vaccine used [23]. This could be due to adherence to traditional practice or fear of financial loss. Additionally, owners may be less willing to vaccinate within the UK due to the lack of severe zoonotic infections, due to the UK being rabies free, and the other viruses not causing any real known issues to human health (although *Leptospira* is zoonotic but rare in the UK). Recent studies by Taylor et al. (2022) [26] showed that around 50% of dogs had at least a single vaccine dose for protection against *Leptospira* given within the 12 months of the study, suggesting that half are missing out on protection against a potentially serious zoonotic disease.

This study found that owners were more likely to have vaccinated their dog in the last 12 months if required by a third party, such as a day care or putting their animal into a dog shelter. This is in line with previous studies that found a higher vaccination rate in dogs staying in boarding kennels where disease risk is often higher due to rapid turnover and close contact with many animals from different backgrounds [23]. Mandatory vaccination has previously been seen in human governance. In the UK, the Vaccination Act of 1853 made it a legal requirement to vaccinate against smallpox, which ultimately led to its eradication [27]. Some may call for a similar move in companion animal vaccination. However, increasing doubts about making childhood vaccinations mandatory and the consequential breakdown of trust between parents and health professionals [28] are also likely to appear with any attempt to impose mandatory vaccination regimes for pets. Indeed, vaccine hesitancy was seen in high levels during the COVID-19 outbreak of 2019–2022, although the reasons why this was the case are poorly understood and require more sympathetic research [9,10,11].

Of particular interest was the mention of autism in dogs post vaccination, with 3.1% (80 people) having “heard that a dog had developed it.” This is interesting given the rapid decrease in MMR vaccination due to concerns that it was linked to autism, as suggested by Andrew Wakefield (now withdrawn). If this has made the jump into veterinary medicine, this could prove highly problematic for vaccine uptake. Although this has been mentioned by people in the past, there is no evidence of the existence of autism in dogs, nor what the clinical signs would be, but this aspect requires further research.

Owner demographics such as having a lower income or living in areas of the UK that are less affluent had no statistical relevance to whether owners vaccinated in the previous year. This is not supported by previous studies that found that vaccination rates were higher in more affluent areas and among those with higher education levels [14,29]. However, the reason for this difference could be due to how data was collected. The use of Facebook groups and voluntary sampling in this study may attract a skewed demographic, whereas the previously mentioned studies collected data from either veterinary electronic health records or through active sampling, which could guarantee them better coverage of certain demographics. However, the UK is currently undergoing a cost-of-living crisis, and thus it is perceived that the level of pet vaccination and other pathogen control methods (anthelminthic and flea treatments) may decrease further, although this remains to be seen. There was a high number of respondents who had received significant education (degree level or higher), and this is likely to reflect that the study was done as part of a post graduate study.

No statistical relevance was discovered regarding the dogs’ demographics. However, 28% of the dogs included were aged two years or less, and 93% of these dogs were vaccinated in the previous 12 months. This follows previous findings that found younger dogs were more likely to be vaccinated [23]. This could be due to owners being more aware of the importance of initial puppy vaccinations for immunity, or, alternatively, when they purchase a puppy it is commonly from a breeder who will likely (if they are reputable) to have had the puppy vaccinated. Owners of older dogs may assume that immunity is lifelong, and they may be correct, as evidence shows that these dogs do hold immunity to core vaccines (CPV, CDV, and CAV 1 and 2) for many years, including, sometimes, the lifetime of the dog [21]. However, this could be due to environmental challenge, which constantly acts to boost the immune system for common viruses such as CPV.

A significant factor that influenced whether an owner chose not to vaccinate their dog in the previous 12 months was that the owners stated they had a lack of time to go to the vet. This suggests that rather than an active political or personal decision to be “anti-vaccination,” for many it is simply a matter of convenience. Studies into why people do not attend their NHS medical heath checks within the UK show that one of the top reasons is having a lack of time or competing priorities [30]. It seems plausible that this would also apply to attending a veterinary appointment outside of emergency situations, especially if the need for vaccination is perceived to be not required. If lack of time is a main concern to keep up to date with required vaccines, a way to make it easier and more convenient for them could be to increase the availability of mobile vets who come to their home or local area to administer preventative health care. This would also be helpful for those owners who vaccinate less due to their dog being stressed by going to the vet or those people with mobility or travelling issues. Implementation of mass rabies vaccination of pet dogs in Africa was conducted via mobile vets in an attempt to control the disease [31], and this was thought to prove relatively successful. They found that a critical need for the success of the program was public education via awareness campaigns featured in different media sources. Targeted education measures have been proven to increase vaccinations rates [32] and may be worth an investment from local veterinary groups or the Department for Environment, Food and Rural Affairs (DEFRA) to encourage more pet vaccine uptake.

This study also found that if people regularly flea treat and worm their dogs as part of a yearly health plan, then they were more likely to have vaccinated their dog in the previous 12 months. This suggests that if a dog’s health care measures are included in a pre-paid package then owners are more likely to vaccinate yearly. Pet health plans are a clever marketing idea that can be useful to encourage dog owners to attend annual health checks. This appears to work well, as visiting a vet was a common reason why pet dogs were vaccinated. There is a concern that if owners see vaccination as the only reason for attending their vet annually, and they choose to not vaccinate, that vets will not see dogs frequently enough to recognize and treat other undiagnosed diseases [20]. It is important to stress to puppy owners the benefits of continued annual check-ups for other health care concerns beyond vaccination. Reminder cards in the post or, more conveniently, a mobile phone app with notifications could be a useful way of helping owners to remember when preventative health care is required for their dog.

In the PAW report, it was found that 28% of surveyed dogs had not received initial puppy vaccinations [14]. This study only found 2% that had never received vaccinations, and the German research only found 1% [23]. This could suggest that even those owners who vaccinate less than recommended have still acquired at least preliminary puppy vaccinations for their dogs and thus are not “anti-vaccination” in mindset but instead possibly “anti-overvaccination.” Anti-overvaccination is different from what is commonly perceived within the “anti-vaccination” community for humans, where vaccine hesitancy means some children are not vaccinated at all. Anti-overvaccination questions whether vaccinating annually or even triennially is safe or required. The WSAVA’s updated guidelines support using triennial serological testing in lieu of revaccination every three years [16]. Dogs who have responded to core vaccinations, not including leptospirosis, should have protective immunity that can be maintained for many years [16,33]. In this study, 13% of respondents stated that they use titer testing. Titer testing is a method that can be used to assess the level of antibodies circulating in a dog’s blood [34]. The presence of adequate serum antibody to declare a protective titer can determine whether that dog requires a booster vaccine or not [35]. Titers for vaccines for leptospirosis are limited because antibodies persist only for short periods [16] and have been found to give only significant protective immunity for 12 months after vaccination [36]. However, leptospirosis, in particular the new Lepto4 vaccine, is a controversial vaccine that has been linked to VAAEs, including allergic events, immune-mediated disease, anaphylaxis, and death [16,37]. However, titer testing results may cost more, and results can take a few days to come back, and therefore people may prefer to vaccinate their pet rather than spend more on a titer test and have to visit a veterinary practice twice in quick succession. This way, it also reduces the requirement for invasive procedures to draw blood for titer testing.

This study found that owners who had heard of negative effects after vaccinating were more likely to titer test if they had not vaccinated their dog in the previous 12 months. In total, 69% of owners stated they had heard of negative side effects due to vaccination, with 16% stating that they had personal experience of them with their dog. In this study, the top five adverse reactions that owners thought could relate to canine vaccination were allergies (37%), lethargy (33%), seizure or convulsions (28%), soreness at the injection site (25%), and autoimmune problems (24%). Previous research on cats has shown that VAAEs negatively affect future vaccination decisions [25]. However, some VAAEs, such as pain, swelling, and redness at the injection site, are considered common [38]; therefore, it can be complicated to differentiate between acceptable VAAEs and those that put into question the safety of that vaccine. According to the Veterinary Medicines Directorate (VMD), in 2019 (the last year where full data is available pre-COVID-19), there were 3578 adverse reaction reports in dogs, which include all drugs administered to dogs, including vaccinations [39].

A retrospective cohort study on adverse events diagnosed within three days of vaccine administration in dogs states that VAAEs have some inherent risk factors and may be generally underreported [40]. Although VAAEs are relatively infrequent in the population, there are certain concerning factors, such as a significant increase of risk with number of vaccines administered per visit and small-breed dogs being at greater risk of a reaction [40]. A large-scale survey conducted in Japan looked at adverse reactions after non-rabies vaccinations. They found a much higher rate of VAAEs than reported in the UK (0.093/10,000 vaccinated dogs) or USA (38.2/10,000 vaccinated dogs) [41]. They also found a higher prevalence of VAAEs in certain small breeds, suggesting a possible genetic predisposition, but admitted that there may have been breed bias with the popularity of small breeds in Japan [41].

It was found that owners who had not vaccinated their dog in the previous 12 months were more likely to titer test if they have worked or studied within the nutrition or vet industry. It seems plausible that owners who express an interest in relevant fields would be more aware of procedures such as titer testing as a recommended and evidence-based safe alternative to vaccinating a dog more often than may be necessary [16]. It could be argued that in future studies on vaccination levels dogs that have been titer tested and proven to have protective immunity should be included in the vaccinated population rather than the unvaccinated population and therefore as contributing towards herd immunity of certain core canine diseases.

Owners who did not vaccinate in the previous 12 months were found to be more likely to titer test if they fed “healthier” dog food or they sought canine diet advice from a nutritionist. These owners would appear to take extra time and effort to find healthier alternatives for their dogs and are willing to research, pay for professional advice, and feed premium dog food products. However, general confusion can be created for dog owners looking for evidence-based information when researching via the internet and even for “experts” in certain fields. Amongst professionals, there is disagreement over certain health movements for dogs, such as, for example, whether raw food diets are dangerous [42,43] or the “gold standard diet for pets” [44]. There are scientists that have criticized the rise of alternative pet medicine and diets, even linking these choices to the anti-vaccination movement itself [45], although there is still much more research to be done in the area of raw food diets and vegan pet diets to assess their safety and benefit (or lack thereof) for pets. It is perhaps worth investigating training non-veterinarian professionals to improve their ability to advise pet owners on areas associated with vaccination and anthelminthic and flea treatments to ensure gold standard treatments are initiated.

Overall, this study found vaccination rates to be higher than previous studies have found. There are limitations to this study, as it was international, and vaccinating protocols vary from country to country. The information provided in the survey is susceptible to the recall bias of each owner. The internet and access to Facebook or Twitter were required in order to take part in the survey. In the past, this may have been a limitation, but recent national statistics in the UK show that 93% of households have access to the internet [46], while 66% of people in the UK own a Facebook page [47].

This survey was referred to as the “Dog Health Care” survey to avoid bias from preconceptions and attitudes towards vaccination. Random dog groups were chosen, rather than targeting dog health groups, to avoid selection bias. However, due to the snowball effect of sharing the survey, it is unknown which Facebook pages or groups the survey was ultimately shared to. Due to the survey being shared on dog-focused Facebook groups, owners with a strong interest in dogs and their care may have been more motivated to take part, resulting in some bias. It has been found that social media sites such as Facebook can create polarized groups with echo chambers, where information with poor evidence goes unchallenged; there are many such sites regarding the vaccination debate [42]. The internet can be a large source of misinformation about topics such as vaccination. This was apparent during the COVID-19 pandemic and the search for a vaccine. The WHO coined the term “infodemic” to describe an “overabundance of information and the rapid spread of misleading or fabricated news, images, and videos. Like the virus, it is highly contagious and grows exponentially and plays a major role in complicating the COVID-19 pandemic response efforts” [48].

There were significantly more female participants than male; therefore, there could also be a gender bias. It has been found women are more likely than men to seek health information online and twice as likely as men to seek material regarding the health of their child [49]. Dogs are treated as and considered part of the family and hold a relationship often like that of a parent–child bond [50]. In modern society, maternal instincts have extended from children to pets, such as dogs [51]; thus, women in particular could be more likely to join Facebook groups dedicated to dogs and respond to a survey regarding their care.

This study has identified factors associated with the likelihood or not of vaccinating dogs within a 12-month period and further factors associated with determining whether someone who has not vaccinated their pet dog in the last 12 months uses titer testing instead. However, with a low incidence of non-vaccination and a high incidence of annual vaccinations, there was no evidence to suggest in this study that dog owners are affected by the anti-vaccination movement identified as causing issues for human immunology and the fight to prevent, control, and eradicate diseases. There does appear to be a rise in concern regarding over-vaccination in dogs, which has been addressed by the WSAVA with updated guidelines for safer vaccination protocols and encouragement to use titer testing triennially. Rather than dog owners being considered “anti-vaccination,” there may be a move towards owners becoming more informed and basing vaccination choices on this.

## 5. Conclusions

This study in the UK investigates issues with vaccine uptake and suggests some factors that merit follow-up research. Further research in the area of canine vaccinations would be helpful to look at whether anti-vaccination attitudes are present, but this study found no evidence that dog owners are avoiding vaccination or hesitant to vaccinate their pets. There may be a tendency towards following WSAVA guidelines and vaccinating less for core vaccines or titer testing. As vaccination rates were lowest in the UK compared to other countries, represented in this study, it would also be interesting to explore internationally what vaccination protocols vet practices are following, as it may be that many still do not follow WSAVA guidelines.

## Figures and Tables

**Table 1:** Vaccination history of participants’ dogs in this study, including vaccination status and frequency when vaccines are given. Note: * indicates multiple answers were possible for the question.

Question	Response Option	Frequency of Responses	Agreement with Reason (%)
**Vaccination in the last 12 months?**	Yes	2034	78.68%
	No	543	21.01%
	Unknown	8	0.31%
	In the UK (1285)	896	69.73%
	In the USA (1041)	926	88.95%
**Certification of vaccination**	Yes	2042	78.99%
	No	440	17.02%
	Unknown	103	3.98%
Vaccination frequency *	Initial puppy vaccinations only	324	12.53%
	Yearly boosters	1597	61.78%
	Every 2–3 years	252	9.75%
	Yearly titer test before vaccinating if required	148	5.73%
	Titer test every few years before vaccinating if required	188	7.27%
	Use of Nosodes instead of vaccines (homeopathic treatment)	24	0.93%
	Never vaccinated	52	2.01%

**Table 2:** Reasons why people vaccinate their pets regularly and awareness of negative effects. Note: * indicates multiple answers were possible for the question.

Question	Response Option	Frequency of Responses	Agreement with Reason (%)
Reasons for vaccinating regularly *	Veterinary advice during consultation	1247	48.24%
	It is included as part of your agreed pet plan with your veterinarian	405	15.67%
	Proof of vaccination required for third party	657	25.42%
	Concern about your dog contracting an infectious disease (such as parvo, distemper, hepatitis, leptospirosis, rabies)	1332	51.53%
	Advice from a dog trainer or behaviorist	73	2.82%
	Advice from friends, family, or other dog owners	126	4.87%
	Internet advice	57	2.21%
	I do not vaccinate regularly when recommended *	537	20.77%
**Heard of negative effects**	Yes	1793	69.36%
	No	792	30.64%
**Experienced negative effects**	Yes	424	16.40%
	No	2161	83.60%

**Table 3:** Negative effects perceived to be due to vaccination people had heard about post canine vaccination. Note: * indicates multiple answers were possible for the question.

Ever Heard of Any of the Following in Connection with Dogs Receiving a Vaccination? *	Frequency of Response	Agreement (%)
Allergies	969	37.49%
Anal gland issues	47	1.82%
Apathy	223	8.63%
Autism	80	3.09%
Autoimmune problems	627	24.26%
Asthma	31	1.20%
Behavioral issues (reactivity/aggression)	431	16.67%
Brain damage	230	8.90%
Broken bones	10	0.39%
Cancer	363	14.04%
Cherry eye (prolapse of the third eyelid gland)	16	0.62%
Conjunctivitis	37	1.43%
Coughing	187	7.23%
Deafness	31	1.20%
Death	511	19.77%
Dementia	34	1.32%
Depression	117	4.53%
Dew claw injury	8	0.31%
Diarrhea	549	21.24%
Ear problems	89	3.44%
Fear/phobias	188	7.27%
Fever	493	19.07%
Fleas	14	0.54%
Gastrointestinal issues	465	17.99%
Hair loss	156	6.03%
Heart issues	105	4.06%
Hip or elbow dysplasia	17	0.66%
Hives	223	8.63%
Lack of appetite	589	22.79%
Lethargy	864	33.42%
Mange	9	0.35%
Meningitis	50	1.93%
Nausea	493	19.07%
Nervousness/anxiety	247	9.56%
Obesity	15	0.58%
Oral ulcers	9	0.35%
Osteoarthritis	23	0.89%
Pain	344	13.31%
Paralysis	179	6.92%
Pica (ingestion of objects that are not food items)	18	0.70%
Seizures or convulsions	735	28.43%
Skin issues (itchy skin, eczema, or dermatitis)	377	14.58%
Soreness at the injection site	659	25.49%
Sneezing	90	3.48%
Stiffness	181	7.00%
Syringomyelia	9	0.35%
Swelling of face or limbs	283	10.95%
Tumors	242	9.36%

**Table 4:** Factors with positive and negative effects on whether an owner vaccinated in the previous 12 months.

Q1—What Factors Are Used to Determine Whether Someone Is Less Likely to Have Vaccinated in the Past 12 Months?					
Response	Reference	*p*-Value	Chi-Square	Odds Ratio	95% CI
**Q7** **_Country of residence**		0.000	25.07		
Aus and NZ	UK			0.72	(0.28, 1.89)
Europe	UK			0.90	(0.38, 2.15)
Other	UK			0.38	(0.08, 1.94)
USA and Canada	UK			0.36	(0.24, 0.54)
**Q9_Dog age**		0.000	52.49	1.21	(1.12, 1.28)
**Q12_Attended adult training classes**		0.014	6.09		
Yes	No			1.65	(1.11, 2.44)
**Q18_Hold a certification of vaccination**		0.000	63.54		
No	Yes			5.97	(3.81, 9.36)
**Q20_I do not feel that vaccines are necessary for my dog**		0.000	26.25		
Yes	No			3.93	(2.29, 6.75)
**Q20_Personal concerns over vaccines causing health issues**		0.000	25.01		
Yes	No			3.03	(1.96, 4.67)
**Q20_Lack of time to go to the vet**		0.008	7.03		
Yes	No			8.48	(1.91, 37.72)
**Q20_I titer test and vaccinate when required**		0.009	6.9		
Yes	No			1.81	(1.16, 2.81)
**Q21_Vaccinate regularly upon veterinary advice**		0.001	11.28		
Yes	No			0.46	(0.29, 0.73)
**Q21_Vaccinate regularly due to requirement of third-party proof**		0.000	18.79		
Yes	No			0.30	(0.16, 0.53)

**Table 5:** Positive and negative factors that affect if an owner has vaccinated in the previous 12 months.

Response	Reference	*p*-Value	Chi-Square	Odds Ratio	95% CI
**Q21** **_Vaccinate regularly due to concern over dog contracting infection**		0.000	25.56		
No	Yes			3.21	(2.02, 5.11)
**Q24_Heard of vaccinations causing autoimmune problems**		0.019	5.49		
Yes	No			1.64	(1.09, 2.48)
**Q24_Heard of vaccinations causing ear problems**		0.013	6.14		
Yes	No			3.36	(1.25, 9.06)
**Q24_Heard of vaccinations causing hair loss**		0.004	8.41		
Yes	No			0.35	(0.17, 0.72)
**Q24_Heard of vaccinations causing soreness**		0.017	5.7		
Yes	No			1.70	(1.10, 2.62)
**Q24_Heard of vaccinations causing swelling**		0.032	4.6		
Yes	No			1.81	(1.05, 3.10)
**Q28_Advice regarding vaccinations received from vet**		0.028	4.82		
Yes	No			0.59	(0.37, 0.94)
**Q30_Guardian worms their dog regularly**		0.000	13.62		
Yes	No			0.47	(0.32, 0.70)
**Q34_Flea and worm regularly due to products being part of pet plan**		0.000	17.44		
No	Yes			7.25	(2.34, 22.47)
**Q35_Feeding raw homemade dog food**		0.015	5.94		
Yes	No			1.78	(1.12, 2.82)
**Q36_Advice received regarding nutrition from other dog owners**		0.036	4.41		
Yes	No			1.56	(1.03, 2.37)

**Table 6:** Positive and negative factors that affect whether an owner who has not vaccinated in the previous 12 months uses titer testing.

Q2. What Factors Are Used to Determine Whether Someone Who Has Not Vaccinated in the Past 12 Months Uses Titer Testing Instead?					
Response	Reference	*p*-Value	Chi-Square	Odds Ratio	95% CI
**Q13** **_Registered at a vet**		0.008	6.94		
No	Yes			0.08	(0.01, 0.81)
**Q15_Neutered or spayed**		0.034	4.48		
No	Yes			0.54	(0.30, 0.96)
**Q18_Hold a certification of vaccination**		0.000	12.82		
No	Yes			0.40	(0.24, 0.67)
**Q20_Vaccinate less as dog finds going to the vet stressful**		0.006	7.64		
Yes	No			0.15	(0.03, 0.74)
**Q20_Vaccinate less due to concern over vaccines causing health issues**		0.027	4.87		
No	Yes			1.81	(1.07, 3.06)
**Q22_Heard of negative effects after vaccinating**		0.001	11.33		
No	Yes			0.13	(0.03, 0.53)
**Q24_Heard of vaccinations causing nausea**		0.002	10.07		
Yes	No			0.39	(0.22, 0.71)
**Q27_Guardian has detoxed their dog**		0.016	5.81		
Yes	No			2.15	(1.15, 4.01)
**Q28_Look for advice regarding vaccines from a professional**		0.001	10.79		
Yes	No			3.30	(1.59, 6.83)
**Q35_Feeding cold-pressed/air-dried/freeze-dried dog food**		0.000	13.27		
Yes	No			3.97	(1.86, 8.50)
**Q35_Feeding raw commercial dog food**		0.000	12.85		
Yes	No			2.49	(1.50, 4.14)
**Q35_Feeding via scavenging or scraps**		0.025	5.05		
Yes	No			0.28	(0.09, 0.92)
**Q36_Advice received regarding nutrition from nutritionist**		0.000	24.63		
Yes	No			3.64	(2.12, 6.16)
**Q36_Education or work in industry vet or nutrition industry**		0.014	6.01		
Yes	No			8.45	(1.33, 53.91)

Odds ratio for response relative to reference response.

## Data Availability

The original contributions presented in this study are included in the article/supplementary material. Further inquiries can be directed to the corresponding author(s).

## Supplementary Materials

The following supporting information can be downloaded at https://www.mdpi.com/article/10.3390/pathogens15070678/s1.

## Abbreviations

The following abbreviations are used in this manuscript:

AIC	Akaike Information Criteria
Aus	Australia
CAV 1	Canine adenovirus 1
CAV 2	Canine adenovirus 2
CDV	Canine distemper virus
CI	Confidence interval
CPV	Canine parvovirus
DEFRA	Department for Environment, Food and Rural Affairs
DOI	Duration of immunity
GDPR	General Data Protection Regulation
MMR	Measles, mumps and rubella
NZ	New Zealand
OR	Odds Ratio
PAW	Animal Wellbeing (PAW) Report
PDSA	People’s Dispensary for Sick Animals
SARS	Severe Acute Respiratory Syndrome
UK	United Kingdom
USA	Unites States of America
VAAEs	Vaccine-associated adverse effects
VGG	Vaccination Guidelines Group
VMD	Veterinary Medicine Directorate
WHO	World Health Organization
WSAVA	World Small Animal Veterinary Association
