# Supplementary material for: Canine Vaccination—A Survey of Owner Attitudes and Adherence to Vaccination Protocols
Source: Pathogens. 2026 Jun 26;15(7):678. doi: 10.3390/pathogens15070678 (PMC13415299; doi:10.3390/pathogens15070678)
Supplement: Supplementary file 1 [file pathogens-15-00678-s001.zip › pathogens-4361268-supplementary.pdf]

Supplementary data

Table S1. Dog descriptor information included in the study including age, breed, where the dog was obtained from, and the level of training which the dog had received. Note * indicates multiple answers were possible for the question.

Question	Response option	Frequency of responses	Percentage of responses
**Dog age**	1 or younger	395	15.28%
	2	324	12.53%
	3	288	11.14%
	4	254	9.83%
	5	221	8.55%
	6	179	6.92%
	7	173	6.69%
	8	164	6.34%
	9	122	4.72%
	10	145	5.61%
	11	100	3.87%
	12 or older	220	8.51%
**Breed**	Pedigree	1535	59.38%
	Mixed Breed	796	30.79%
	Designer mix	254	9.83%
**Obtained**	Professional breeder	792	30.64%
	Rehome or private rescue	91	3.52%
	Adoption shelter/ charity	869	33.62%
	Amateur breeder (friend/ Family Member)	279	10.79%
	Internet website (e.g. dogs4sale, preloved or gumtree)	174	6.73%
	Stray dog found	38	1.47%
	Pet store	19	0.74%
	Puppy farm	14	0.54%
	Home-bred	140	5.42%
	Not stated	169	6.54%
**Training acquired ***	Self-trained	2113	81.74%
	Group puppy classes	1060	41.01%
	Group adult dog classes	894	34.58%
	Private training with a dog trainer or behaviorist	733	28.36%

Table S2. Age groups, gender, education levels, employment status and annual earnings of people included within this study.

Question	Response option	Frequency of responses	Percentage of responses
**Age of owners**	18-39 years	1151	44.53%
	40-59 years	1046	40.46%
	Over 60 years	388	15.01%
**Gender**	Female	2457	95.05%
	Male	104	4.02%
	Prefer not to say/ other gender	24	0.93%
**Education**	University higher degree (e.g. MSc, PhD)	467	18.07%
	University degree level (e.g. BSc, BA, PGCE, graduate diploma)	1003	38.80%
	Diploma of higher education or Foundation degree	282	10.91%
	AS or A levels or equivalent (e.g. access to higher education diploma, NVQ's, BTEC diploma)	264	10.21%
	GCSE's or equivalent (e.g. NVQ, apprenticeship, Level 1 & 2 diploma, O-levels	199	7.70%
	Other vocational/ work-related qualifications	129	4.99%
	Foreign qualifications	109	4.22%
	Prefer not to say/ No qualifications	132	5.11%
**Employment status**	Employed, working full time (over 35 hours)	1191	46.07%
	Employed, working part time (under 35 hours)	356	13.77%
	Self-employed/ freelancing	439	16.98%
	Student	108	4.18%
	Retired	307	11.88%
	Unemployed, looking for work	44	1.70%
	Unemployed, not looking for work	52	2.01%

	Not able to work	61	2.36%
	Prefer not to say	27	1.04%
**Annual earnings**	Under £20K	693	26.81%
	Between £21k - £40k	604	23.37%
	Between £41k – £60k	324	12.53%
	Between £61k – £80k	160	6.19%
	Between £81K - £100k	101	3.91%
	£101K or over	107	4.14%
	Prefer not to say	596	23.06%

Table S3. Reasons for worming and flea treatments given to dogs, and information regarding diets. Note * indicates multiple answers were possible for the question.

Question	Response option	Frequency of responses	Agreement with question (%)
**Reasons for regularly treating?***	Veterinary advice during consultation	1320	51.06%
	Products are included as part of your yearly pet plan with your veterinarian	297	11.49%
	Concern regarding your dog's health if they have fleas or worms	1488	57.56%
	Prevention of the spread of fleas or worms to your home, friends, family, other pets	1432	55.40%
	Advice from a dog trainer or behaviorist	61	2.36%
	Advice from friends, family or other dog owners	113	4.37%
	Internet advice	63	2.44%
	I do not treat regularly	332	12.84%
	Proof of treatment required for third party	26	1.01%
**Dog Food ***	Extruded dry	875	33.85%
	Baked dry	978	37.83%
	Cold pressed/ air/ freeze dry	302	11.68%
	Wet food	683	26.42%
	Raw – homemade	392	15.16%
	Raw – commercial	588	22.75%

	Fresh commercial foods	143	5.53%
	Homemade cooked diet	372	14.39%
	Scavenging or scraps	186	7.20%
	Specialist diet	20	0.77%
**Where look for advice on nutrition? ***	Your veterinarian during consultation	1365	52.80%
	Advice from a professional (trainer, behaviorist, food company, pet store, groomer, breeder)	405	15.67%
	A dog nutritionist	556	21.51%
	The internet	996	38.53%
	Your friends or family	292	11.30%
	Other dog owners	660	25.53%
	Personal research (books/ articles/ journals)	1394	53.93%
	Personal experience	20	0.77%
	Education or work in vet or nutrition industry	21	0.81%

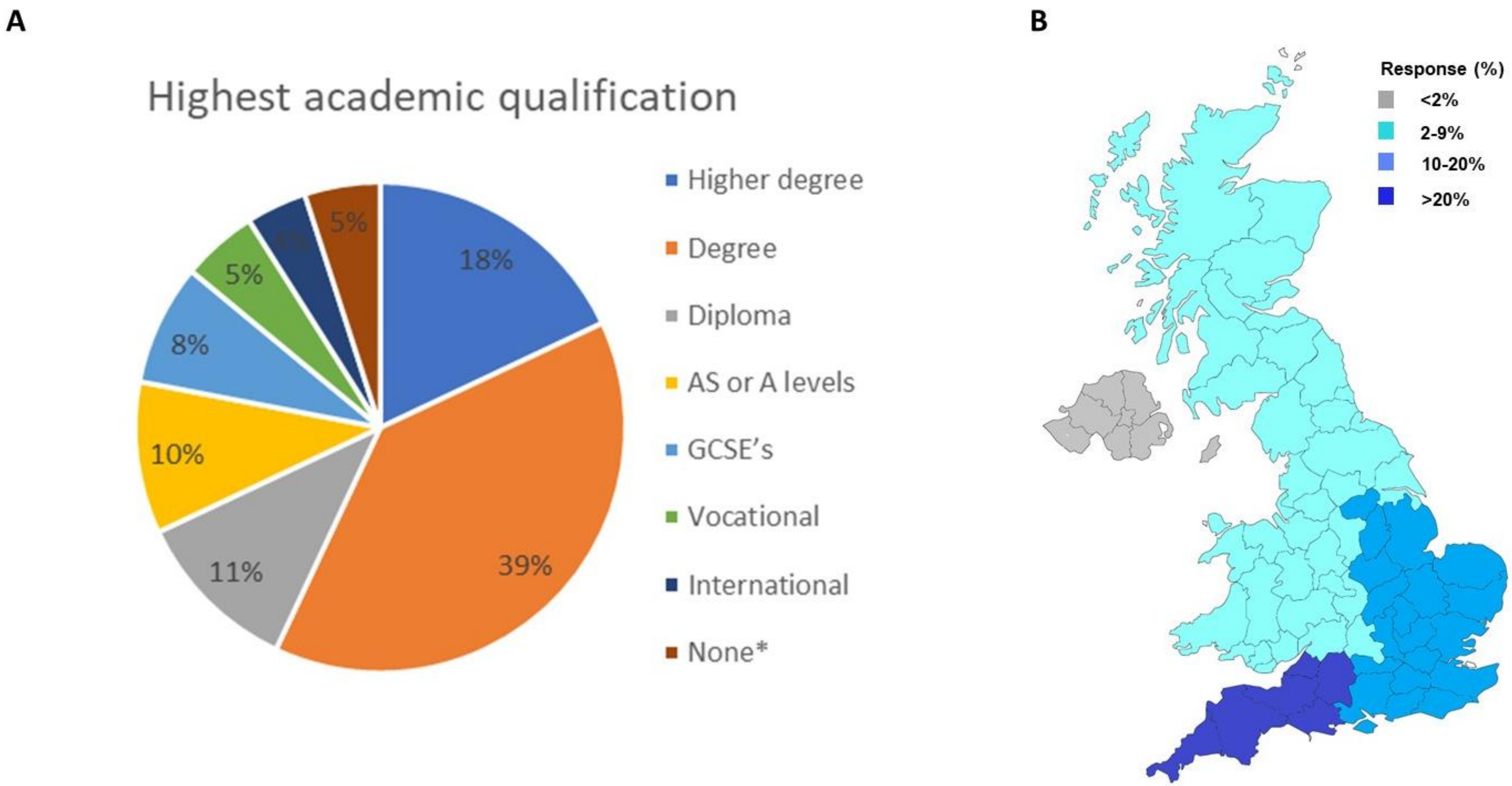
A
Highest academic qualification
18%
39%
11%
10%
8%
5%
5%
Higher degree
Degree
Diploma
AS or A levels
GCSE's
Vocational
International
None*
B
Response (%)
<2%
2-9%
10-20%
>20%

Figure S1. A. education level of the respondents within this study, and B approximate regional location of the respondents to the study questionnaire within the UK where appropriate based on their indication of location on a provided UK map.

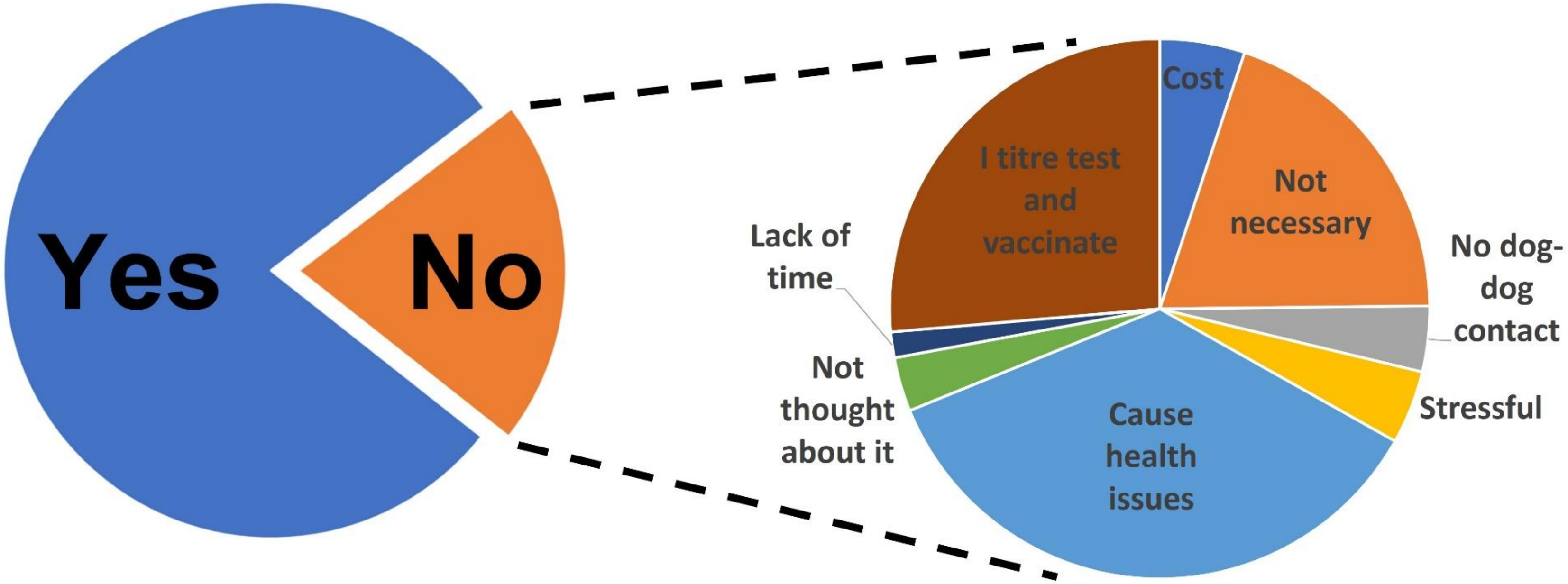
Yes
No
Cost
Not necessary
No dog-dog contact
Stressful
Cause health issues
Not thought about it
Lack of time
I titre test and vaccinate

Figure S2. Reasons why people do not vaccinate their pets.

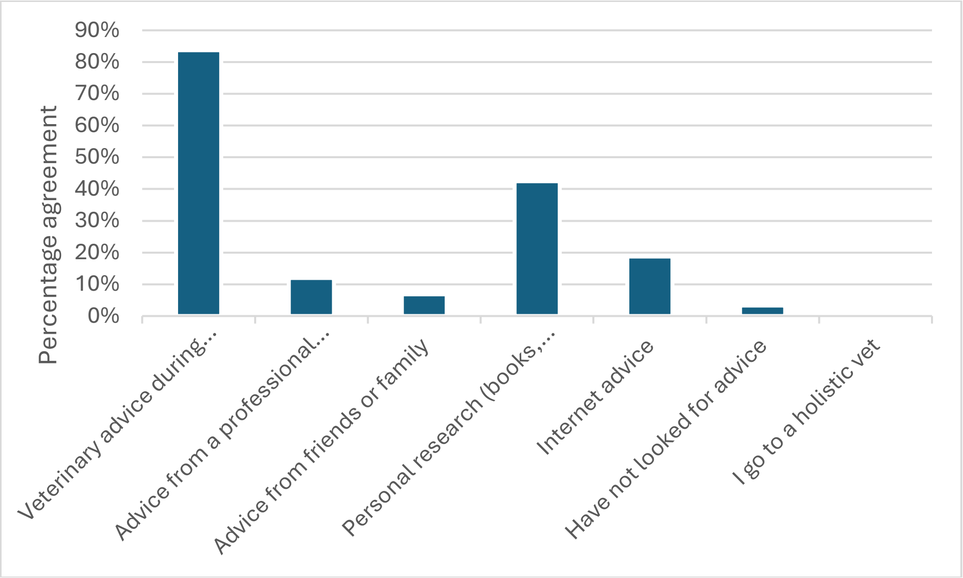

Figure S3. Where do owners get advice from regarding vaccination, and have they tried other methods of vaccination such as detox. Note * indicates multiple answers were possible for the question.

Supplementary data - Questionnaire

If you have multiple dogs at home, please answer the questions in reference to the dog you know best. Remember to answer all the questions with this dog in mind.

OWNER INFORMATION

Q1. What is your age?

Under 18 years
18-39 years
40-59 years
Over 60 years

Q2. What is your gender?

Male
Female
Non-binary/ third gender
Prefer to self-describe
Prefer not to say

Q3. What is the highest level of education you have achieved?

GCSE's grades D- G or equivalent (e.g. NVQ, Level 1 diploma)
GCSE's grades A*- C or equivalent (e.g. NVQ, apprenticeship, Level 2 diploma, O-levels
AS or A levels or equivalent (e.g. access to higher education diploma, NVQ's, BTEC diploma)
Diploma of higher education or Foundation degree
University degree level (e.g. BSc, BA, PGCE, graduate diploma)
University higher degree (e.g. MSc, PhD)
Other vocational/ work-related qualifications
Foreign qualifications
No qualifications
Prefer not to say

Q4. Which of the following categories best describes your employment status?

Employed, working full time (over 35 hours)
Employed, working part time (under 35 hours)
Self-employed/ freelancing
Unemployed, looking for work
Unemployed, not looking for work
Retired
Student
Not able to work
Prefer not to say

Q5. How much do you earn per year?

Under £20K

Between £21k - £40k
Between £41k – £60k
Between £61k – £80k
Between £81K - £100k
£101K or over
Prefer not to say

Q6. What is your current country of residence?

………………………..

Q7.If you live in the UK, what is the first two letters in your postcode? e.g. if your postcode is AB12 3CD, then just write AB

………………………..

DOG INFORMATION

Q8. What is the age of your dog in months and years? (if you are unsure please provide an approximation)

………………………

Q9. What breed is your dog?

……………………..

Q10. Where did you obtain your dog from?
Home-bred
Professional breeder
Amateur breeder (friend/ Family Member)
Adoption shelter/ charity
Internet website (e.g. dogs4sale, preloved or gumtree)
Pet store
Puppy farm
Other …………

Q11. Have you trained with your dog by using one of the following means? Select all that apply.
Self-trained at home
Group puppy classes
Group adult dog classes (including agility/ trick training/ obedience/ rally/ sports etc)
Private training with a dog trainer or behaviourist
Q12. Is your dog currently registered at a vet?
Yes
No

Q13. Is your dog insured?
Yes
No

Q14. Is your dog neutered or spayed?
Yes

No

Q15. Is your dog microchipped?
Yes
No

VACCINATION INFORMATION

Q16. Has your dog been vaccinated in the last 12 months?
Yes
No
Unknown

Q17. Have you any certification concerning your dog's vaccination status?
Yes
No
Unknown

Q18. Since you acquired your dog, how often do you vaccinate?
Yearly boosters
Every 2-3 years
Yearly Titre test before vaccinating if required
Titre test every few years before vaccinating if required
Use of Nosodes instead of vaccines (homeopathic treatment)
Initial puppy vaccinations only
Never vaccinated

Q19. If you vaccinate less frequently than recommended by your vet, what are the reasons for this? Select all that apply.
Cost of vaccination
I do not feel in my opinion that vaccines are necessary for my dog
My dog does not come into contact with other animals
My dog finds it stressful going to the vets
Personal concerns over vaccines causing health issues
Have not thought about it
Lack of time to go to the vets
Not registered at any vet
I vaccinate regularly when recommended
Other ………………

Q20. If you vaccinate regularly when recommended by your vet, why is this? Select all that apply.
Veterinary advice during consultation
It is included as part of your agreed pet plan with your veterinarian
Groomers, day cares or boarding facilities require proof of vaccination
Concern about your dog contracting an infectious disease (such as Parvo, Distemper, Hepatitis, Leptospirosis, Rabies)
Advice from a dog trainer or behaviourist
Advice from friends or family
Advice from other dog owners
Internet advice
I do not vaccinate regularly when recommended

Other …………….

Q21. Have you ever heard of dogs having negative effects after vaccinating?
Yes
No

Q22. Have you ever experienced your dog having a negative side effect after vaccination?
Yes
No

Q23. Have you ever heard of any of the following health concerns in connection with dogs receiving vaccinations? Select all that apply.
Allergies
Anal gland issues
Apathy
Autism
Autoimmune problems
Asthma
Behavioural issues (reactivity/ aggression)
Brain damage
Broken bones
Cancer
Cherry eye (prolapse of the third eyelid gland)
Conjunctivitis
Coughing
Deafness
Death
Dementia
Depression
Dew claw injury
Diarrhoea
Ear problems
Fear/ phobias
Fever
Fleas
Gastrointestinal issues
Hair loss
Heart issues
Hip or elbow dysplasia
Hives
Lack of appetite
Lethargy
Mange
Meningitis
Nausea
Nervousness/ anxiety
Obesity
Oral ulcers
Osteoarthritis
Pain
Paralysis

Pica (ingestion of objects that are not food items)
Seizures or convulsions
Skin issues (itchy skin, eczema or dermatitis)
Soreness at the injection site
Sneezing
Stiffness
Syringomyelia
Swelling of face or limbs
Tumours

Q24. Are you concerned that any of the above health issues may have occurred as a result of vaccination for your dog? If so which.
……………………………

Q25. Have you heard of detoxing your dog after receiving a vaccination?
Yes
No

Q26. If yes, have you ever detoxed your dog after a vaccination?
(Such as the use of probiotics, homoeopathy or micro-algae such as 'chlorella')
Yes
No

Q27. Where do you go for advice about vaccinations? Select all that apply.
Veterinary advice during consultation
Advice from a dog trainer or behaviourist
Advice from friends or family
Personal research (books, articles, journals)
Internet advice
Have not looked for advice
Other …………………..

OTHER HEALTH CARE INFORMATION

Q28. Do you keep up to date with your dog's flea treatments?
Yes
No

Q29. Do you keep up to date with your dog's worming treatments?
Yes
No

Q30. Where do you get your flea and worming treatments from? Select all that apply.
Your veterinarian
Shop bought
Internet veterinary stores (e.g. Viovet)
Internet shopping (e.g. Amazon)
Other……………………………

Q31. Have you ever 'heard of' negative side effects due to either flea or worming treatments?
Yes

No

Q32. Have you 'experienced' your dog having a negative side effect due to either their flea or worming treatments? If so, please state what this was below.

…………………………………….

Q33. If you treat your dog for fleas and worms regularly, what are your reasons for doing so? Select all that apply.
Veterinary advice during consultation
Products are included as part of your yearly pet plan with your veterinarian
Concern regarding your dog's health if they have fleas or worms
Prevention of the spread of fleas or worms to your home, friends, family, other pets
Advice from a dog trainer or behaviourist
Advice from friends or family
Advice from other dog owners
Internet advice
Other ……………………………..

Q34. What do you feed your dog? Select all that apply.
Extruded dry
Baked dry
Cold pressed/ air/ freeze dry
Wet food
Raw – homemade
Raw – commercial
Fresh commercial foods
Homemade cooked diet
Scavenging or scraps
Other ……………………………….

Q35. Where do you look for advice regarding nutrition? Select all that apply.
Your veterinarian during consultation
A dog trainer or behaviourist
A dog nutritionist
The internet
Your friends or family
Other dog owners
Personal research (books/ articles/ journals)
Other
